# Real-Time Decoding of Brain Responses to Visuospatial Attention Using 7T fMRI

**DOI:** 10.1371/journal.pone.0027638

**Published:** 2011-11-14

**Authors:** Patrik Andersson, Josien P. W. Pluim, Jeroen C. W. Siero, Stefan Klein, Max A. Viergever, Nick F. Ramsey

**Affiliations:** 1 Image Sciences Institute, University Medical Center Utrecht, Utrecht, The Netherlands; 2 Rudolf Magnus Institute of Neuroscience, Department of Neurology and Neurosurgery, Division of Neuroscience, University Medical Center Utrecht, Utrecht, The Netherlands; 3 Biomedical Imaging Group Rotterdam, Department of Radiology and Medical Informatics, ErasmusMC, Rotterdam, The Netherlands; French National Centre for Scientific Research, France

## Abstract

Brain-Computer interface technologies mean to create new communication channels between our mind and our environment, independent of the motor system, by detecting and classifying self regulation of local brain activity. BCIs can provide patients with severe paralysis a means to communicate and to live more independent lives. There has been a growing interest in using invasive recordings for BCI to improve the signal quality. This also potentially gives access to new control strategies previously inaccessible by non-invasive methods. However, before surgery, the best implantation site needs to be determined. The blood-oxygen-level dependent signal changes measured with fMRI have been shown to agree well spatially with those found with invasive electrodes, and are the best option for pre-surgical localization. We show, using real-time fMRI at 7T, that eye movement-independent visuospatial attention can be used as a reliable control strategy for BCIs. At this field strength even subtle signal changes can be detected in single trials thanks to the high contrast-to-noise ratio. A group of healthy subjects were instructed to move their attention between three (two peripheral and one central) spatial target regions while keeping their gaze fixated at the center. The activated regions were first located and thereafter the subjects were given real-time feedback based on the activity in these regions. All subjects managed to regulate local brain areas without training, which suggests that visuospatial attention is a promising new target for intracranial BCI. ECoG data recorded from one epilepsy patient showed that local changes in gamma-power can be used to separate the three classes.

## Introduction

In any interactions with our environment, including speech, we fully depend on the motor system. Damage to neurons involved in motor control can restrict this ability or even completely disrupt communication between our mind and our environment, as in the case of locked-in-syndrome [1]. Situations such as loss of motor function in severe paralysis would greatly benefit from additional means of interaction. By measuring cortical activation changes and linking these changes to commands one can “outsource” the muscular control to a computer and create new channels through which intentions can be transmitted. These techniques are commonly referred to as Brain-Computer-Interfaces (BCI) [2], [3].

Because of its availability and non-invasiveness EEG has been the predominant modality in BCI research. To reach the extra-cranial electrodes the neural electrical potentials have to go through the cerebrospinal fluid, dura mater, skull and scalp. In effect, the signals lose power, bandwidth and spatial resolution. By implanting electrocorticographic (ECoG) or intracortical microelectrode arrays one can record signals much more specific in both time and space, and with a much higher signal-to-noise ratio (SNR), compared to EEG. Encouraged by the success in non-human primates [4]–[7], there is a growing interest in applying intracranial technologies for human BCI [8]–[13]. Because the dominating modality in BCI research has been EEG, the control strategies investigated, also for invasive measurements, have mainly been based on systems located in cortical areas accessible by scalp electrodes. The most common strategies have been P300 responses [14]–[16], steady state visual evoked potentials (SSVEP) [17]–[19] and motor imagery [9], [10], [20]. While these types of control have been shown to work in both healthy subjects and patients, many studies have reported that part of the study population is not able to learn control even after training [21]–[25]. Moreover, patients might have clinical issues making these strategies inapplicable. It is for example uncertain whether paralysed people are capable of engaging their motor cortex after a long period of non-use [26]. This indicates that in the light of intracranial solutions, alternative avenues, using other brain systems, are worth exploring to further the BCI field and to be able to create an individually optimized setup for each patient. While the term “BCI illiteracy” is sometimes used for subjects not able to control a BCI it is more likely that the particular control task is not suitable and that by choosing the right task also these subjects can learn to gain control.

Intracranial electrodes make it possible to access brain functions that are located deeper in the brain or are otherwise inaccessible for EEG.

Here we present a new avenue for intracranial BCI, which exploits specific properties of the visual system. With the help of attention we can select what sensory information to focus processing resources on [27]. Covert visuospatial attention, i.e. focusing attention on a specific part of the visual field in order to better process what happens in this spatial region while maintaining gaze at the center of the field, is known to induce changes in activity in the visuospatial cortex [28]–[33].

Two earlier studies have examined the potential use of brain activity associated with covert visual spatial attention for BCI control, and demonstrated that changes in the alpha band could be detected using MEG [34] or EEG [35]. The induced changes could be classified with offline techniques, but as realtime analysis was not tested it is not clear whether these changes offer enough detail for BCI application. Spatial detail of MEG and EEG may be a limiting factor in exploiting the brain activity patterns associated with covert attention. The retinotopy assures that attention to a restricted part of the visual field corresponds to activity in restricted cortical areas. An intracranial BCI system with high resolution, based on e.g. ECoG, should be able to react only to local attention and not to attention anywhere in the peripheral visual field. More attention target regions can be added to increase the degrees of freedom. Moreover, an attention target region could be moved to the place in the visual field mapped to the cortical region most suitable for implantation.

In principle, functional MRI yields better detail compared to MEG and EEG, at the expense of speed of detecting changes. Moreover, it is inherently sensitive to activity anywhere in the brain, and as such can be used to investigate new alternative control tasks and cortical regions. Real-time fMRI [36]–[40] offers the possibility of identifying target regions for intracranial electrode placement presurgically and can be used to train the patient beforehand. Although fMRI measures bloodflow as opposed to electrical or magnetic signals, fMRI activations have been shown to agree with those found with ECoG [13], [41]–[43]. Spatial correlations between activity patterns obtained with both has been shown to be particulary strong in the high gamma band (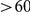
Hz) [43]. The use of real-time fMRI for learned self-regulation of local brain activity has been demonstrated several times before [40]. Most of these studies have had a neurofeedback approach, where the self-regulation was not investigated with the purpose of transmitting commands. Here the feedback was given directly on changes in the BOLD signal. Building on these results, the technique has also been applied for BCI purposes where the signal changes are classified to discrete outputs representing intentions (see review in [44]). Activity induced by covert attention is rather subtle and for the present purpose of realtime decoding, requires the most sensitive fMRI technique available. Ultra-high field MRI systems have become available recently, and have been shown to yield excellent sensitivity [45]. To test our hypothesis we implemented real-time fMRI on a 7 Tesla MR scanner using healthy volunteers. We postulate that if realtime decoding is feasible with covert attention and fMRI, placement of electrodes on the visual cortex should also yield decodable signals. We also report on decodability of ECoG signals obtained from visual cortex in a patient undergoing neurosurgery for epilepsy.

## Materials and Methods

**Figure 1:**
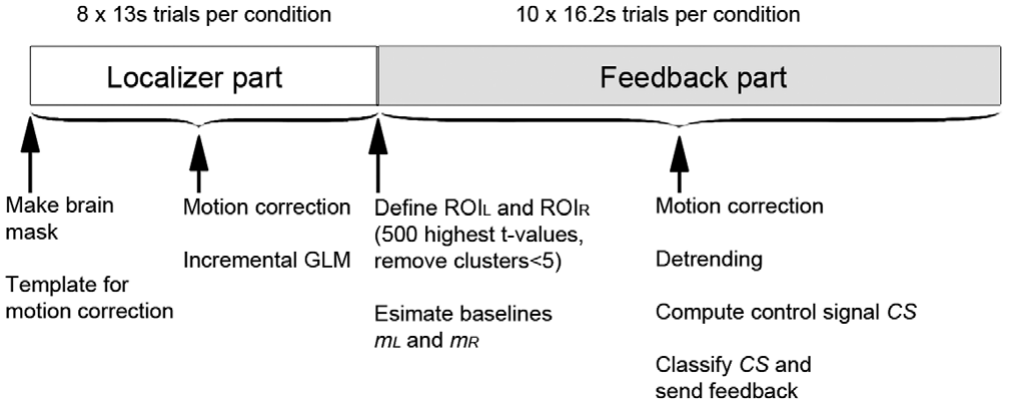
Timeline of the experiment. The localizer and feedback data are acquired in the same fMRI run.

The experiment was performed in a single fMRI run in which the healthy volunteers were instructed via a central cue to move their attention to one of three target regions while maintaining their gaze at the center. The scan consisted of two parts; a first part in which we located the activated regions and a second part in which subjects were given real-time feedback based on the activity in these regions. An overview of the full experiment can be seen in Figure 1.

### Subjects

fMRI data were acquired from ten healthy volunteers (age 19-27, 6 female, all except one right handed). One of the subjects showed very poor performance during the experiment. After the experiment the subject communicated problems with concentration and offline inspection of the fMRI data showed excessive motion. Based on this we have excluded this subject. Two additional subjects performed the task outside the scanner while we recorded their eye movements using electrooculography (EOG).

Multi-channel subdural ECoG data was recorded from one patient (female, age 26, left hemisphere) undergoing neurosurgery for epilepsy.

The protocol was approved by the ethics committee of the University Medical Center Utrecht in accordance with the declaration of Helsinki (2008), and all subjects had given their written informed consent. All subjects were naive to the task.

### fMRI data acquisition and real-time system

The data were collected on a 7T Philips Achieva system with a 16-channel headcoil. The functional data were recorded using an EPI sequence (TR/TE = 1620/25 ms; FA = 90; SENSE factor = 2; 35 coronal slices, acquisition matrix 96x96, slice thickness 2mm with no gap, 1.848 mm in-slice resolution). The FOV was selected so it covered the occipital lobe. A total of 500 volumes were acquired in a single run and divided into 200 volumes of localizing relevant brain areas (localizing part) and 300 volumes of real-time feedback based on activation in these located regions (feedback part). Directly following reconstruction on the scanner the data were sent to a separate computer performing the analysis (Dual-Core 2.5 GHz notebook) via the local network using a TCP/IP protocol and the Philips DRIN (Direct Reconstruction INterface) module. The stimulus was projected to the subject from a second computer via a video projector. An update-trigger containing information about the direction and color of the instruction marker was sent to the second computer via a serial cable. Except for the motion correction all the parts were implemented in Matlab (Mathworks, Natick, MA).

### Task

**Figure 2:**
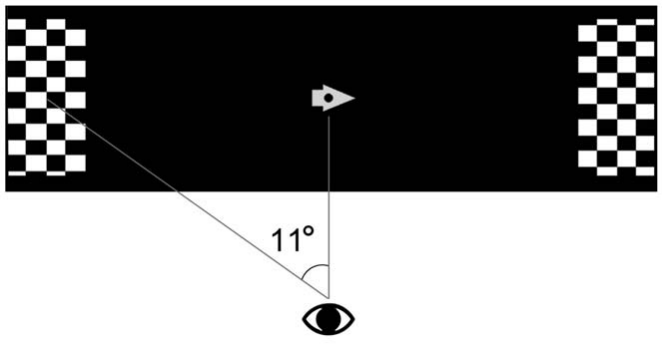
The visual stimuli.

The visual stimuli were constructed as two rectangular areas, one in the left peripheral visual field and one in the right, each containing a checkered pattern and both at a visual angle of 11 degrees relative to a central cue (Figure 2). To facilitate the shifting of attention direction, we made the checkered patterns scroll (2s per cycle) upwards on the right side and downwards on the left. In the center was a marker on which the subjects were instructed to fixate their gaze at all times. Both checkerboards were constantly visible throughout the fMRI runs, while the center marker was alternated between a right arrow, a left arrow and a circle. The arrows indicated to which side the subject had to direct the visual attention. The circle indicated that the attention was to be directed to the center. The three trial types were repeated in a pseudo random scheme with the restriction of no two adjacent attention trials being in the same direction.

### Localizing part

#### Trials

The localizing part consisted of eight trials of each condition plus one extra initial central attention trial, each being eight scans (13.0 seconds) long. The instruction was updated first after the analysis finished (1.0 seconds on average). This time has been accounted for in all plots and results.

#### Motion correction

The first volume was used as the template for motion correction and all the subsequent volumes were aligned to it using a rigid transformation. The registration was performed by minimization of the sum of squared differences between grey-value intensities. To achieve real-time performance, a stochastic gradient descent method [46] was employed for optimization, using 50 iterations. The images were blurred with a Gaussian filter (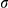 = 1 voxel) prior to image registration. Linear interpolation was used during optimization while cubic B-spline interpolation was used to generate the final rotated/translated image. The algorithm was implemented in C++, and called from Matlab. The computation time was approximately 0.6s per fMRI volume.

#### Analysis

To find the activated voxels in real time we implemented the incremental GLM method described in [47]. The incremental approach ensures that the computation time does not grow with the number of scans. By keeping the whole experiment in a single run we minimize the risk of movement between selection of ROIs and the feedback experiment and we get an improved estimation of the low frequency drift and therefore a better detrending and a better control signal. Three regressors representing right- and left-sided attention, and a linear function as a simple model for the drift were included in the model. Since visual spatial attention induces both increased BOLD signal in retinotopically mapped regions and decreased signal in unattended regions [31], [48]–[50], the differential contrasts “right-left” and “left-right” were used when computing the t-maps. This also made sure we avoided picking up regions responding to attention in general.

#### ROI selection

When the localization part was finished (200 volumes) the resulting t-maps were used for making the two sets of voxels representing right versus left side attention and left versus right side attention (denoted 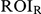 and 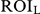 respectively) as follows. First the two t-maps were masked to only include values inside the brain. The mask was constructed by first thresholding a smoothed image volume and then filling any holes. The two most anterior of the coronal slices were excluded from the mask to exclude boundary artifacts from the registration. For each of the two t-maps the voxels with the 500 highest t-values were selected and from these clusters smaller than five voxels were removed. The remaining sets of voxels constituted the 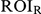 and 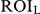. Next, a baseline value was computed for each ROI, 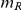 and 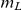, by averaging the signal inside the ROI in the data recorded during the central condition. The first three volumes (4.86s) in central trials that were preceded by an attention trial were excluded to let the signal return to baseline. Additionally, the individual time series of the voxels making up the ROIs were saved for the purpose of detrending during feedback.

### Feedback part

#### Trials

During feedback a longer trial of 10 scans (16.2 seconds) was used, and each condition was repeated 10 times. Feedback was given by coloring the central instruction marker according to the performance (see *Classification and feedback*). As during the localizer part, the instruction was updated after the analysis (0.8 seconds on average). Also here we have accounted for this delay in all results.

#### Analysis

In the feedback part of the scan we gave the subjects real-time information about their performance based on the activity in 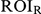 and 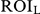, as follows. When a new volume was available it was first motion-corrected as during the localizing part. After this the values inside the two ROIs were extracted and added to the time series of available data (including the localizer part). To remove any low frequency drift [51] in the signal, detrending was now applied using an algorithm originally described in the context of real-time detrending of heart-rate variability measurements [52] (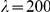). Each voxel' time series was detrended individually since the signal drift looked quite different in different parts of the image. The new detrended values were averaged to give a single value per ROI and fMRI volume (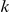), 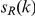 and 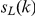. These numbers were in turn normalized to a percentage change from the baseline and subtracted to give the value of the control signal 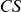 defined as
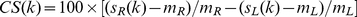
(1)where 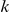 is the volume number and 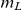 and 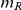 are the baseline values computed from the localizer data.

#### Classification and feedback

The control signal was classified based on its magnitude using three thresholds above the baseline (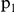, 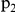 and 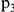) and three below (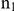, 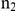 and 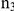). The central instruction marker was then colored according to this classification. During attention two tones of green represented weak and strong signals in the correct direction whereas two tones of red represented a control signal indicating the wrong (or lack of) direction (see Figure S1). During the central condition green represented a signal close to baseline. For Subjects 1-7 fixed 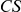 thresholds of 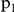 = 1.5, 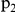 = 2.5, 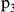 = 4, 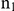 = -1.5, 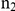 = -2.5, 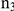 = -4 were used. These values turned out to be rather conservative, and for Subjects 8-9 an adaptive thresholding approach was applied, where the localizing data were used to select individual values online. First a retrospective 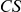 was computed applying Equation 1 to the available (localizer) data. Then, for both right and left attention, the thresholds required to limit the false positive rate (FPR) to 0.2 were estimated. These estimated thresholds were used as 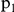 and 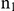. Here we needed a binary classification and for right attention the value of 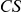 was classified as ’positive’ if larger, and ’negative’ if smaller than 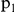. Thus, the FPRs were computed using false positives from both the other conditions, i.e. opposite and center attention. In order to account for the hemodynamic delay, the instructions were shifted 3 TRs with respect to the control signal before computing the FPR. The other threshold levels were now set as 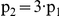, 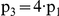, 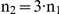, 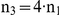.

### Performance

The true positive rate (TPR) and false positive rate (FPR) were used as a measure of performance. As when determining the adaptive thresholds, the instructions were shifted 3 TRs to account for the hemodynamic delay. The FPR was computed both including and excluding the central condition. The reason for considering only the attention blocks is that the BOLD undershoot following an attention block may produce a rebound in 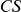 towards the opposite side of the baseline. This is a BOLD effect and would not be present in a BCI based on electrophysiological measurements, e.g. EEG. Hence, to give a fairer measure of stability during attention, the FPR was also computed after removing the ’attend center’ blocks. To visualize how the TPR and FPR depended on the thresholds, they were computed for varying threshold levels and the results were plotted as receiver operating characteristic (ROC) curves.

The performance depends on the thresholds 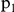 and 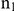, and since only Subjects 8 and 9 were classified using adaptive thresholding, we also recomputed the performance for Subjects 1 to 7 offline applying the same adaptive method.

### Offline group analysis

For the group analysis we used SPM5. Each subject's realigned data were normalized to the Montreal Neurological Institute (MNI) space using the structural T1 image. The normalized functional images were smoothed with an isotropic 4mm FWHM Gaussian kernel and then used to compute activation patterns. The second level analysis was performed using a paired t-test (attend left, attend right) on the resulting beta images and the contrasts right-left and left-right were applied.

### ECoG data and analysis

The patient had a 64-channel (8×8) electrode grid positioned on the left parietal-occipital cortex, covering a considerable part of the cortex included in the fMRI volume for the healthy volunteers. Data were collected during a localizer task (20 trials attend left, 20 attend right, 39 attend center, no feedback), with 5 s trial duration. The signal was acquired at 512 Hz, and was referenced to a common average across all 64-channels. The first 4 seconds (after instruction) of each trial were used to compute the power in the high gamma band (65–95 Hz). This single band was chosen as fMRI matched this frequency range in previous studies [13], [43], [53]. Performance was estimated by means of a leave-one-out cross-validation approach. For each trial, all the other trials (constituting a “training set”) were used to create a classifier. Each classifier was a simple linear combination of channels (electrodes), resembling the fMRI approach.

Each of the 79 cross-validation tests was performed in three steps; (1) Normalization, (2) Selection of channels and (3) Classification of the test trial.

1. All channels (including the test trial data) were normalized, to zero mean and unit variance, as estimated using the 78 training trials.
2. Three sets of channels were identified, one for each attention direction. Each set contained the channels where the average amplitude differed enough between the corresponding direction and the other two. For a channel to be included in one of the sets it should; 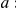 have an average higher (lower, if negative due to deactivation) during this attention direction than for the other two, with a difference to the closest one larger than a certain threshold 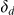 (see *Optimization*). 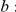 have an average during this attention direction with a value exceeding half the standard deviation computed over all three directions. Note that a channel can be selected for two of the attention directions if there is an increase in gamma power during one direction, and a decrease in the other.
3. The test trial was classified as the direction whose set of channels had the highest average magnitude.

#### Optimization

To optimize the performance, the selection of channels was computed for a range of thresholds (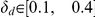), each giving a different selection. For each of these selections the training trials were themselves classified, as described above. The final selection, used for classifying the test trial, was the one giving the most correct classifications of the training data.

## Results

### Control signals

**Figure 3:**
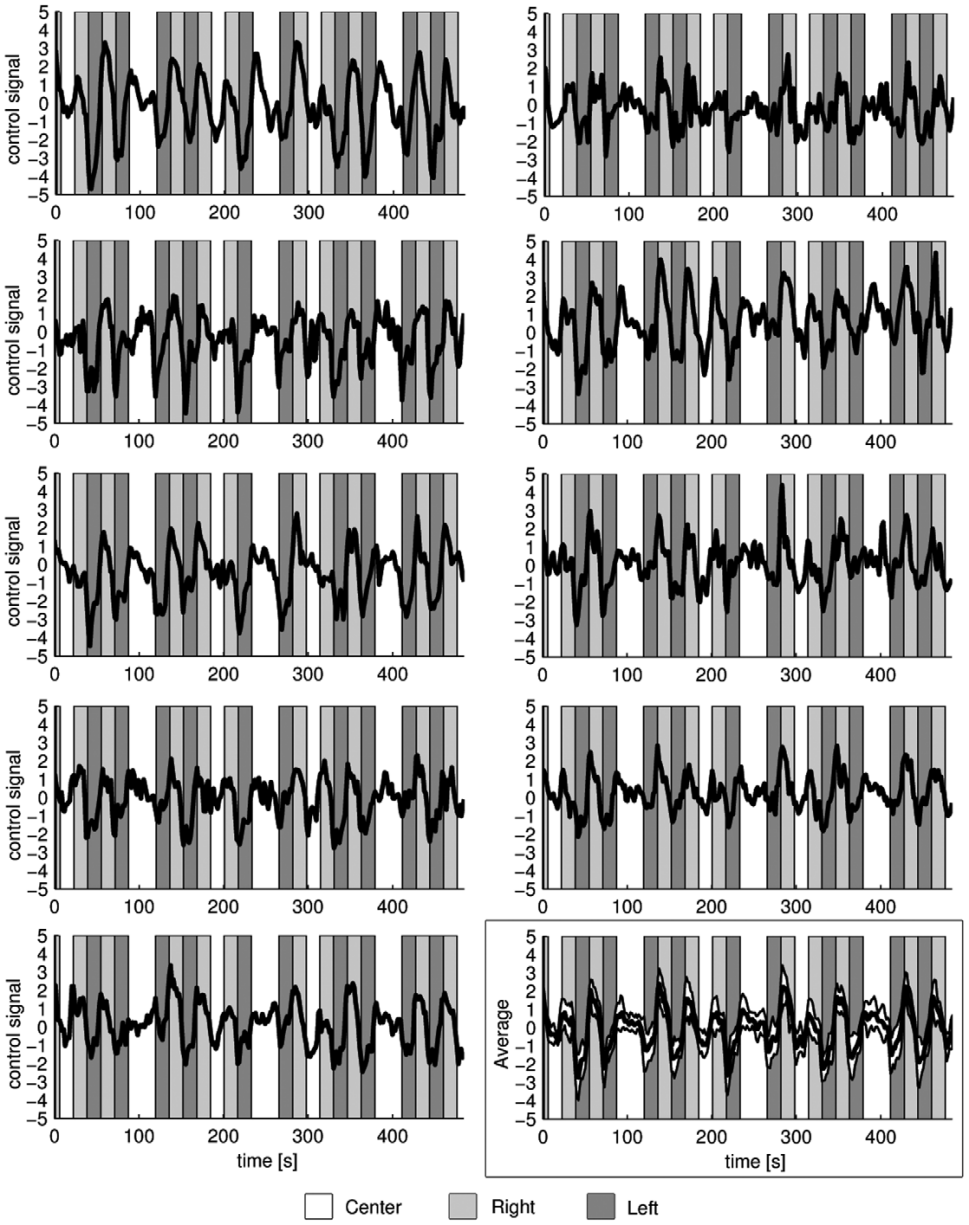
The control signals (CS) for all subjects. (Subject 1-9 from left to right and top to bottom.) Light and dark gray represent right-sided and left-sided attention respectively. The blocks have been shifted 3 TRs (4.9s) to compensate for the hemodynamic delay. The last plot shows the average control signal over all subjects, with the standard deviation shown in white.

**Figure 4:**
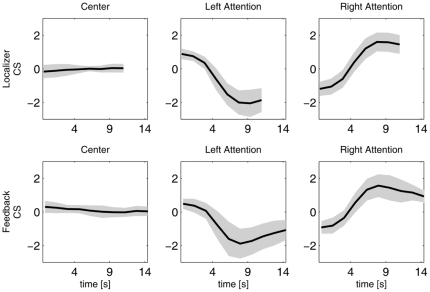
Average control signal during the central, right-sided attention and left-sided attention trials. The averages are shown both for the actual control signal during feedback and the control signal computed offline using the localizing data. The standard deviation is shown in gray.

The control signals (CS) for all subjects are plotted in Figure 3. Dark gray, light gray and white represent the left attention, right attention and center conditions, respectively. The condition blocks are shifted 3 TRs (4.9 s) to compensate for the hemodynamic delay. The responses to the different conditions were also averaged, first for the individual subject, then over all nine subjects. The results are plotted in Figure 4.

The strength of the attention-modulated signal changes in 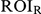 and 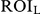 relative to their baselines might not be equal. This means that when subtracted (see Equation 1), CS may be biased towards one of the directions. Such an effect can be seen in Figure 3 for Subjects 3 and 5. This bias can in turn lead to a difference between the two sides in the time needed to exceed the thresholds. A more laterally symmetric control signal, and one that is more uniform across subjects, could be achieved by normalizing the signals using both the baseline and the standard deviation as 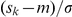 (see Equation 1).

### Performance online

Table 1 shows the true positive rates (TPR) and false positive rates (FPR) from the online results. For Subjects 1 to 7 fixed CS thresholds of 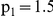 and 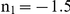 were used. The low number of true positives together with the near absence of false positives indicates that these thresholds were rather conservative.

**Table 1:** Online performance.

	TPR		FPR		FPR†	
Subject	L	R	L	R	L	R
1	0.64	0.39	0.02	0.07	0.00	0.00
2	0.28	0.13	0.03	0.00	0.00	0.00
3	0.55	0.08	0.04	0.01	0.01	0.00
4	0.17	0.54	0.02	0.09	0.00	0.02
5	0.68	0.21	0.00	0.00	0.00	0.00
6	0.18	0.29	0.02	0.01	0.00	0.00
7	0.40	0.11	0.01	0.00	0.00	0.00
Average	0.41	0.25	0.02	0.03	0.00	0.00
8	0.73	0.81	0.19	0.29	0.09	0.13
9	0.77	0.89	0.18	0.31	0.05	0.16
Average	0.75	0.85	0.19	0.30	0.07	0.15

Online True Positive Rate (TPR) and False Positive Rate (FPR). Fixed thresholds of 1.5 and -1.5 were used for Subjects 1 to 7, whereas adaptive thresholding was applied to Subjects 8 and 9 (see Table 2). (L  =  left attention, R  =  right attention,

† excluding the ’attend center’ condition.)

For Subjects 8 and 9 adaptive thresholding was applied. The localizer data were used here to estimate what threshold levels are needed to restrict the FPR to 0.2. In this way we could increase the number of true positives, while estimating the risk.

### Performance offline

To assess what the performance would have been if we had applied the adaptive thresholding to all subjects we recomputed the analysis offline for Subjects 1 to 7. In this analysis the thresholds were based on the localizer data in the same way as was done online for Subjects 8 and 9. Table 2 shows the new thresholds together with the resulting TPR and FPR values.

**Table 2:** Offline performance.

	threshold		TPR		FPR		FPR†	
Subject	L	R	L	R	L	R	L	R
1	-0.27	0.22	0.89	0.78	0.28	0.20	0.13	0.03
2	-0.32	0.16	0.90	0.67	0.29	0.14	0.13	0.01
3	-0.52	0.20	0.91	0.69	0.21	0.20	0.13	0.00
4	-0.27	0.37	0.61	0.94	0.12	0.36	0.00	0.18
5	-0.34	0.40	0.99	0.53	0.26	0.13	0.26	0.00
6	0.01	0.31	0.82	0.73	0.30	0.23	0.17	0.06
7	-0.06	0.18	0.89	0.88	0.24	0.23	0.09	0.07
Average			0.86	0.75	0.24	0.21	0.13	0.05
8	-0.15	0.22						
9	-0.24	0.18						

Offline True Positive Rate (TPR) and False Positive Rate (FPR) for Subjects 1 to 7 when applying adaptive thresholding. Columns 2–3 show the corresponding thresholds. We also incuded these numbers for Subjects 8 and 9 where the method was applied online. (L  =  left attention, R  =  right attention,

† excluding the ’attend center’ condition.)

**Figure 5:**
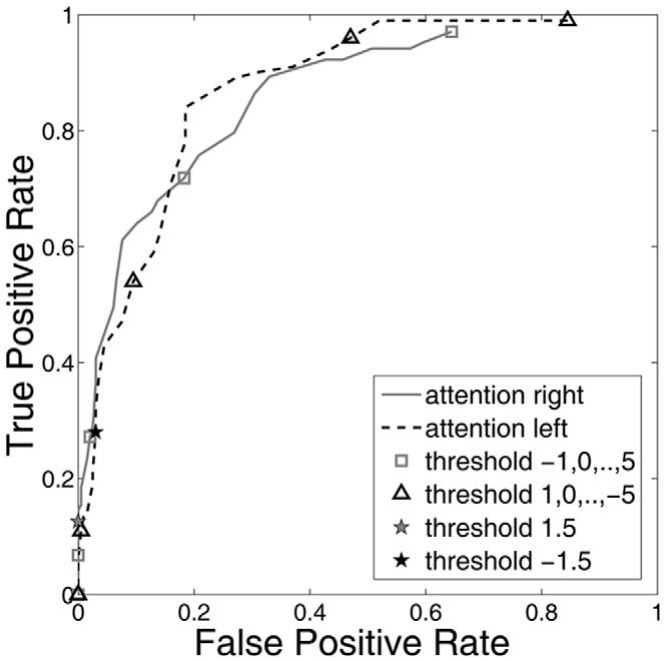
ROC curves plotted for the control signal classification of Subject 2 over varying thresholds.

A more detailed view of how the classification depends on the thresholds for Subject 2 is given by the ROC curves in Figure 5 (all subjects' ROC curves are plotted in Figure S2). The TPR and FPR were computed using thresholds between -1 and 5 for right-sided and 1 and -5 for left-sided attention. The unit thresholds are marked in the plots as squares and triangles and the levels for the online thresholds, 1.5 and -1.5, are indicated by stars. Note that these values were not used online for Subjects 8 and 9.

**Figure 6:**
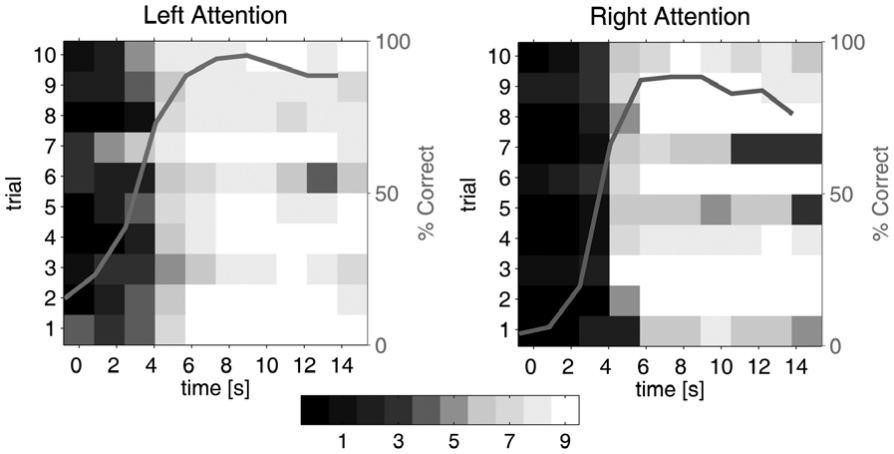
Number of subjects having a specific image volume correctly classified. Each row represents one of the 10 trials, and each column a time point (not adjusted for hemodynamic delay) in that trial. The curves show, for all time points, how many trials would be correctly classified if based only on this particular volume.

An overview of the classification results over the different trials is presented in Figure 6. For each time point (not adjusted for hemodynamic delay) it shows the number of subjects with a correct classification. We also computed the percentage of all trials, for all subjects, that would be correctly classified if based on a single volume. The curves in Figure 6 show the results for classification based on each of the 10 time points within the trials. Classifying the trials using only the 5th time point gives an average correct classification of 89% for left attention and 88% for right attention.

Though the RT-fMRI setup presented here is not meant to be directly used as a BCI, but rather as a tool to practice and evaluate control tasks, a bit rate can be computed. The most commonly used bit rate definition in the field of BCI is the one from Wolpaw [54]. This definition assumes that the classification accuracy is the same for all classes and that the errors are equally distributed. To fulfil these requirements we excluded the center class so that each left and right attention trial were assigned to either left or right. When each trial was classified using only the fifth time point the average accuracy was 92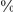 (left 93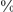, right 91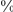), with the increase due to having no false negatives from the central attention class. With each trial being 16.2 seconds this gave a bit rate of 2.2 bits/minute. This number should not be seen as a highest possible bit rate using a two direction visual attention task. Based on a direct measure, e.g. ECoG, the time needed to make a classification will be much shorter.

### ROI selection

The t-maps from the online analysis of the localizing data were thresholded to the two ROIs, 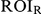 and 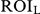. Off-line inspection showed that the t-values corresponding to the 500 voxels threshold were between 2.56 and 4.85 (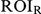; mean 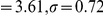, 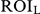; mean 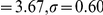). (Table S1 shows the individual values for both 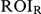 and 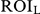 as well as the size of the final ROIs, i.e. after removing clusters smaller than 5 voxels.)

### Incremental GLM analysis

The incremental GLM makes it possible to do the whole experiment in a single fMRI run. The alternative is to stop after the localizer data have been collected to do the statistical analysis and define the ROIs, and then restart to do the feedback part. Offline comparisons show that the incremental method [47] gives an end result very similar to a standard ’full data’ GLM analysis using the same regressors and contrasts. ROIs were for the latter method computed as online, but based on t-maps computed from the full localizer data set at once, instead of in incremental steps. These ’full data’ ROIs, 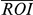, were then compared to the incremental ROIs, 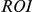, using the Dice coefficient computed as
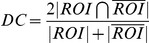
where 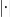 is the volume.

The average numbers across subjects, 0.98 for 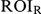 and 0.99 for 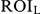, indicate an almost perfect overlap and suggest that using the online incremental GLM does not decrease the sensitivity.

### Group analysis

**Figure 7:**
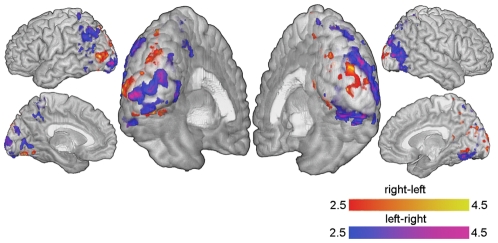
The group activation pattern. Red represents t-values from the contrast ‘attend right-attend left’ while blue represents ’attend left-attend right’.

**Figure 8:**
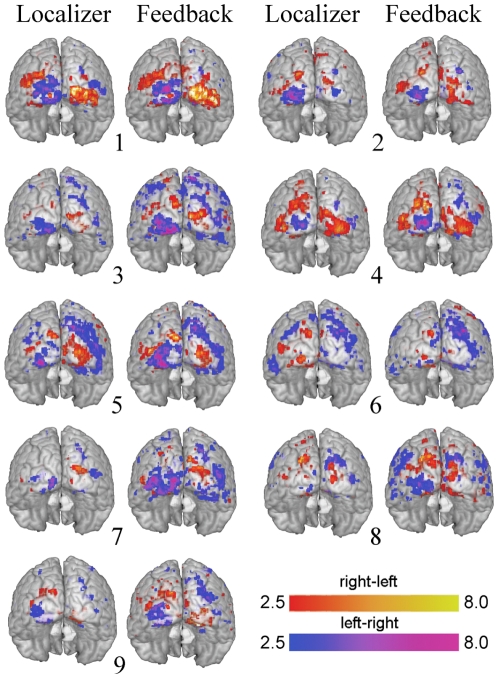
The individual subjects'activation patterns. The patterns both during the localizer part and the feedback part are displayed on the MNI brain. The red and blue color scales represent t-values from the contrasts ’attend right minus attend left’ and ’attend left minus attend right’.

To find the most frequently activated cortical regions during the two attention conditions a group analysis was conducted. The t-maps from the second-level analysis are displayed in Figure 7. The activation patterns for all individual subjects (transformed to MNI space) are displayed in Figure 8 both for the localizing and the feedback data. Figure S3 shows the group distribution of voxels selected for the ROIs, projected on transversal slices.

**Figure 9:**
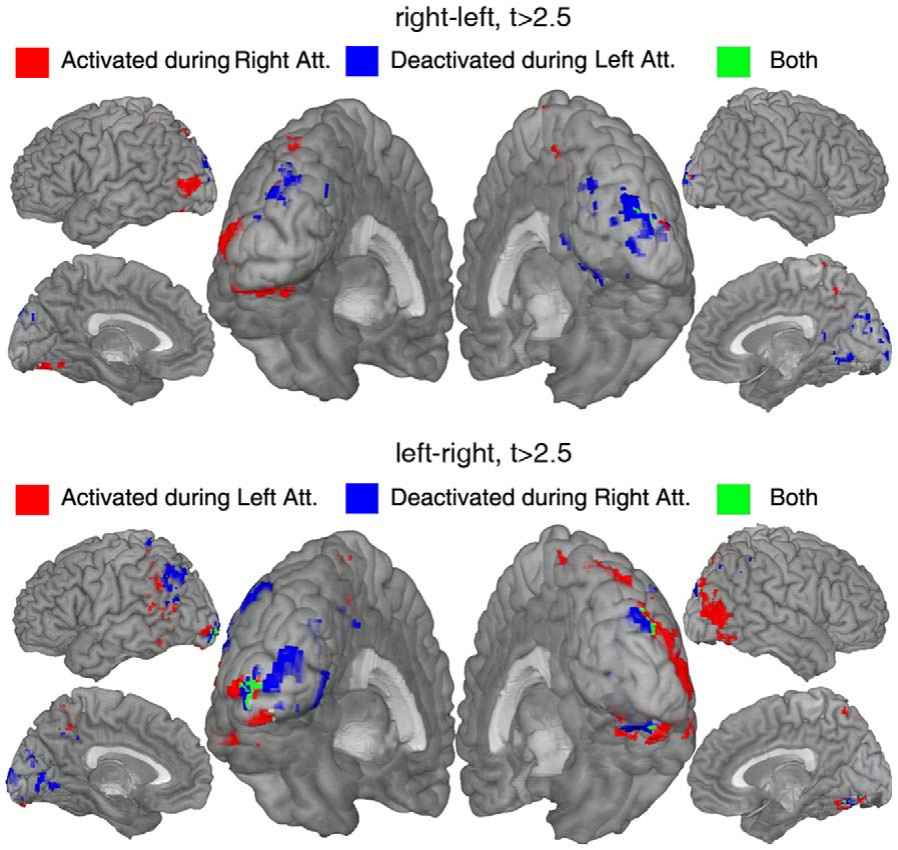
Activations and deactivations. The group t-values higher than 2.5 (see Figure 7) are separated into areas showing activation versus areas showing deactivation relative to the central attention task. The upper half shows the contrast ’attend right-attend left’ and the lower half ’attend left-attend right’. Red represents voxels whose contribution comes from increased activity, blue the voxels showing deactivation during attention to the other side, and green voxels showing both these effects.

The contrasts, and therefore the control signal, are sensitive to both activation during attention to one side and deactivation during attention to the opposite side, i.e. a high t-value for ’right-left’ can be due to increased activity during right attention or decreased activity during left attention, or both. Figure 9 separates the areas in Figure 7 into voxels contributing to the differential contrasts by means of positive activation and voxels whose contributions come from a deactivation during opposing attention. An interesting effect can be seen in the foveal regions around the occipital poles in Figure 7 and Figure 9. These regions show deactivation during contralateral attention. A possible explanation could be that part of the visual field between center and attended periphery is suppressed to reduce interference.

### Eye movements

Any eye movements correlated to the instructions could induce activations falsely interpreted as attention related. If these regions end up in the ROIs it would mean that the regulatory control would partly be based on motor activity. Even though it has been shown multiple times that people have no trouble performing covert spatial attention shifts in the absence of any eye-movements (e.g. [30], [32], [34]) we decided to test subjects’ abilities to perform the task while maintaining a central fixation. Without an eye-tracker approved for use at 7T, we could not record the eye movements during the experiment. Instead we had two additional subjects, naive to the task and not part of the rest of the study, performing the task outside the scanner during which we recorded their eye movements using electrooculography (EOG) with two electrodes below and lateral to the right eye, and a reference electrode behind the ear. These subjects showed no eye movements correlated to the task. (Figure S4 shows the average EOG response in both electrodes for one of the subjects.)

The activity patterns themselves can also be used as an indication of whether or not eye-movements were present. If the gaze is moved to fixate on one of the targets, this target will move to the center of the visual field while the instruction cue, to which the subject will now have to move the attention in order to notice new instructions, and the opposing target will be located in the contralateral hemifield. Since each hemifield is represented by the contralateral visual cortex this would mean that, except for the foveal region, only the ipsilateral side would be activated. In other words, if the subjects moved the gaze to the targets instead of keeping it fixed at the center, the “left attention” condition would only show activity in the right hemisphere and vice versa. This was confirmed for Subject 8 in an additional localizing run where the subject was asked to move the fixation the checkerboards. When compared to the pattern seen during covert attention, the result is distinctly different and laterally mirrored (see Figure S5).

If the subjects instead made small saccades towards the target and back, the BOLD signal changes would not have been strong enough for us to classify them in single images.

### ECoG

**Figure 10:**
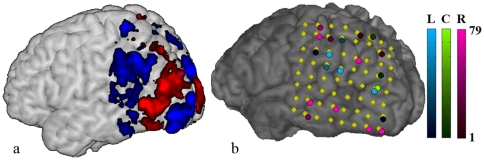
ECoG electrode selections. (a) The fMRI group activation pattern (t 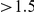, red: ’right-left’, blue: ’left-right’). (b) The yellow markers show the electrodes' locations on the cortical surface. On top of the markers it is shown in how many of the leave-one-out tests the electrode was included. Red, blue and green represent right, left and center attention.

The average TPR over the 79 cross-validation tests was 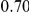 (right: 0.55, left: 0.60, center: 0.82). It should be noted that almost half of the trials were center attention. Figure 10b shows the number of times an electrode was selected to be included in the classifier for one of the leave-one-out tests, and for which class. The yellow markers show the locations of the electrodes, and the colored circles the selection frequency. The locations of the selected electrodes can be compared to the fMRI groupmap in Figure 10a.

## Discussion

In this study we show that brain signals associated with covert visuospatial attention can be used for BCI control. Unique to this approach is that the user can process information in the central visual field while simultaneously exerting control over a device merely by directing attention to the peripheral field.

The brain activation patterns confirm earlier studies on visuospatial attention, but are here decoded in real-time. Our subjects easily managed to avoid eye movements during the task. The results, together with the fact that fMRI activations have been shown to agree with those found with intracranial electrodes [13], [41]–[43], have direct implications for BCI implants. A high performance across subjects and activation confined to a few small brain areas, suggest that the new control paradigm is well suited for intracranial implants.

Using a 7 Tesla MRI system we show that signals from the visual cortex are highly correlated to the direction of visual attention, and can be reliably decoded in real time. One could argue that the use of moving checkerboards introduces a confound by inducing activity due to visual motion (e.g. in area V5). However, we scanned two subjects using stationary stimuli (simple triangles in the periphery) and found the same activation pattern and performance (TPR; 90%/80% and 80%/80% for left/right attention). Further, by using the checkerboard stimuli we show that real visual input would not necessarily affect the attention-based control signal. Thus, even in real-life situations with input covering the full visual field our attention-based BCI approach is likely to work, although this requires further testing.

### Cortical activations

Visuospatial attention, i.e. attention to central or peripheral parts of the visual field while maintaining gaze to the center, has been shown to cause region-specific changes in brain activity as measured with fMRI [29]–[33], [48], [55], [56]. An important finding was the close topographical match of regions activated by actual visual stimuli and by mere attention. Topography of the visual field on the visual cortex has been elucidated in great detail [57]. In V1 and in the encircling areas V2 and V3 each hemifield maps onto the contralateral hemisphere, and stimuli above and below the horizontal meridian are mapped onto the ventral and dorsal regions respectively. From the center of the visual field to the periphery, cortical representations are laid out from the occipital pole towards more anterior aspects of the visual cortex. The parts of the visual cortex that correspond to the attended region exhibit an increased BOLD signal during directed attention, also in the absence of a visual input or eye movements [48], [58]. Importantly, while brain areas processing the attended location exhibit an increased BOLD signal, a decreased signal is seen in brain areas responsible for the part of the visual field surrounding the attended location [48]–[50] and for locations containing distracting elements [31].

Activation patterns in the present study show that the BOLD changes occur in the expected parts of the visual cortex. We find activation in anterior regions of the contralateral occipital cortex, which corresponds to the location of the attended checkerboard. Since the checkerboard crosses the horizontal meridian, both the dorsal and ventral parts of V1-V3 are activated. Though V1 activation is often found on an individual level it is relatively weak and the effect is washed out in the group analysis, see Figure 7. Without a full retinotopic mapping we can not know for certain which visual areas correspond to the activation clusters, but the activations close to the posterior part of intraparietal sulcus (Figure 7 and Figure 8) are likely V3A and/or V3B.

Almost all subjects showed activity at the ipsilateral occipital pole (Figure 8), a region representing the foveal part of the visual field. Figure 9 informs us that the effect in this region is a deactivation during contralateral attention, i.e. the right occipital pole gets suppressed during left peripheral attention. The same effect was reported by Brefczynski-Lewis et.al. [33].

Though the overall pattern was the same across subjects there were also variations, both in location and size of activation clusters. This is partly due to the fact that the anatomical locations and sizes of the visual field maps vary across individuals [57], [59], [60], but on top of this there is also an individual variation in the attentional topography, e.g. amount of ipsilateral effect and the spread of the activation [33]. However, the individual pattern is consistent and does not change over sessions [33] which is important when considering BCI and implantation of electrodes.

### Control signals and classification

The fixed thresholds used for classification in subjects 1-7 turned out to be very conservative, resulting in most images being classified as ’off’. For those subjects the average TPR were 0.41 and 0.25 for left and right, respectively. The adaptive thresholding applied to the two other subjects greatly improved the online sensitivity, while still limiting the false positives. With this improvement, these subjects’ TPR averaged 0.75, for left, and 0.85, for right. This motivated an offline re-computation of the first group's performance using the same adaptive method, increasing the TPR to 0.86 and 0.75 for left and right, respectively.

Besides these numbers, based on individual images, we computed a measure of performance by classifying each complete trial. However, since the aim was to test the stability of our control paradigm and its capacity in the context of implanted electrodes, not to optimize the BOLD classification, we avoided time averaging of the data. Instead we also classified each trial using only the 5th time point. This still gave an average correct classification of 89% for left attention and 88% for right attention (Figure 6).

These numbers are in the upper range of what has been reported with EEG based systems using e.g. motor imagery and SSVEP [61], [62]. It should be noted that we have included an ’off’ class (central attention), which in practice makes it a three-class paradigm. The inclusion of a inclusion of a ’no-choice’ option is something that is often overlooked in BCI studies [63]. If we would have classified each time point using only the options of left or right attention, the performance would have been even higher.

Classification of fMRI data is inevitably slow since the BOLD response has a delay of around 5 seconds after neural firing, and it takes a long time before the signal returns to baseline. However, the time delay will not be present in a true BCI system based on electrophysiological signals. Naturally, a quick detection is desired also for our purpose of task evaluation and subject training, but here the few seconds delay is more acceptable.

### Suggested improvements

The thresholds should be estimated online as was done for two of our subjects. In this way one can take advantage of the individual differences. The ROI selection can be improved in several ways. As a starting point, we used a fixed number of 500 voxels to include in each ROI. However, the number of voxels selected should probably not be a fixed value but somehow depend on the t-value distribution. On the other hand, a fixed t-value threshold could lead to unpredictable results due to a large variation of the ROI sizes across subjects. It would also be possible to put anatomical restrictions on the ROIs. By defining a mask based on a structural image the voxel selection can be restricted to e.g. a single hemisphere.

### Potential

We have shown that the BOLD response following a covert shift of attention to a peripheral region in the visual space is strong enough to be classified in a single trial. Although BOLD is an indirect measure of neural activity, the spatial locations identified by fMRI have been shown to closely match those found using invasive electrophysiological measurements [13], [41], [42]. Despite a limited number of trials and the non-optimal placement of the electrode grid, our ECoG data show that it possible to classify the same attention task using the power in the gamma band. Hence, it is likely that signals recorded by electrodes placed at the optimal positions, as located by fMRI, can be classified with at least the accuracy of our fMRI system. Moreover, the detection will be much quicker based on the electrical response, compared to when using the hemodynamic response.

The spatial attention strategy has some attractive features not found in the tasks commonly used for BCI, such as motor imagery. First, the degrees of freedom can be increased by simply adding more peripheral target regions. Second, a target region can be moved to the location in the visual field that is mapped to, and activates, the cortical area most suitable for implantation. It is also possible that by using this property, and selecting to activate a superficial brain area, it will be easier to pick up the signal changes with EEG or fNIRS.

The real-time fMRI setup described here can be used for evaluating new paradigms as potential control tasks, and to train subjects in them. When planning implantation of intracranial electrodes, the BCI setup can be tried out before surgery in order to locate the best and most stable positions.

## Supporting Information

Figure S1 **Table displaying the colors used for performance feedback.**
(EPS)

Figure S2 **ROC curves plotted for the control signal over varying thresholds.** (Subject 1– from left to right and top to bottom.)
(EPS)

Figure S3 **The number of subjects having a voxel included in an ROI.** The red scale represents ROI_R_ and the blue scale ROI_L_. Due to interpolation during normalization, the numbers are not integers.
(EPS)

Figure S4 **Eye movements.** The plots show the eye movements for one of the two subjects (not part of the rest of the study) measured using EOG outside the scanner while performing the localizer task. Two electrodes, E1 and E2, were placed below and lateral to the right eye, respectively, and were referenced to an electrode placed behind the ear. The dotted line shows the response level during actual eye movements to the target regions (two lines for E2 since the response to the two directions has opposing polarity.).
(EPS)

Figure S5 **The difference in activation pattern between covert attention and actual directed gaze.** The localization part of the experiment was repeated for Subject 8 with the instruction to direct the gaze to the target. The overlay show t-values > 3 for the contrasts ’right-left’ and ’left-right’.
(EPS)

Table S1 **T-value thresholds and ROI sizes.** T-values corresponding to the threshold of 500 voxels used to define the ROIs. |ROI| is the number of voxels in the final ROI, after removing all clusters smaller than five voxels.
(PDF)
