# Supplementary material for: Real-Time Decoding of Brain Responses to Visuospatial Attention Using 7T fMRI
Source: PLoS One. 2011 Nov 14;6(11):e27638. doi: 10.1371/journal.pone.0027638 (PMC3215723; doi:10.1371/journal.pone.0027638)
Supplement: Table S1 — T-value thresholds and ROI sizes. T-values corresponding to the threshold of 500 voxels used to define the ROIs. |ROI| is the number of voxels in the final ROI, after removing all clusters smaller than five voxels. (PDF) [file pone.0027638.s006.pdf]

Subject	t ROI_R	$\lvert ROI_R \rvert$	t ROI_L	$\lvert ROI_L \rvert$
1	4.71	456	3.89	367
2	2.99	397	3.03	389
3	2.85	293	3.70	389
4	4.49	420	2.85	330
5	4.36	410	4.85	397
6	3.45	439	3.66	425
7	2.56	331	3.03	384
8	3.36	428	4.26	363
9	3.71	297	3.76	260
